# Impacts of upstream drought and water withdrawals on the health and survival of downstream estuarine oyster populations

**DOI:** 10.1002/ece3.291

**Published:** 2012-07

**Authors:** Laura E Petes, Alicia J Brown, Carley R Knight

**Affiliations:** 1 Climate Program Office, National Oceanic & Atmospheric AdministrationSSMC3, 1315 East-West Highway, Silver Spring, Maryland 20910; 2 Florida State University Coastal & Marine Laboratory3618 Highway 98, St. Teresa, Florida 32358; 3 Gulf Coast Research Laboratory, University of Southern Mississippi703 East Beach Drive, Ocean Springs, Mississippi 39564

**Keywords:** Apalachicola, climate change, Dermo disease, drought, estuary, oyster, water

## Abstract

Increases in the frequency, duration, and severity of regional drought pose major threats to the health and integrity of downstream ecosystems. During 2007–2008, the U.S. southeast experienced one of the most severe droughts on record. Drought and water withdrawals in the upstream watershed led to decreased freshwater input to Apalachicola Bay, Florida, an estuary that is home to a diversity of commercially and ecologically important organisms. This study applied a combination of laboratory experiments and field observations to investigate the effects of reduced freshwater input on Apalachicola oysters. Oysters suffered significant disease-related mortality under high-salinity, drought conditions, particularly during the warm summer months. Mortality was size-specific, with large oysters of commercially harvestable size being more susceptible than small oysters. A potential salinity threshold was revealed between 17 and 25 ppt, where small oysters began to suffer mortality, and large oysters exhibited an increase in mortality. These findings have important implications for watershed management, because upstream freshwater releases could be carefully timed and allocated during stressful periods of the summer to reduce disease-related oyster mortality. Integrated, forward-looking water management is needed, particularly under future scenarios of climate change and human population growth, to sustain the valuable ecosystem services on which humans depend.

## Introduction

The global availability of fresh water is under threat from both climate change and human population growth. Climate change is altering patterns in precipitation, leading to increases in the frequency and severity of drought events (IPCC 2007). Drought-related water shortages are compounded by human population growth and increased extractions to meet municipal and agricultural demands (Vörösmarty et al. 2000). The global water crisis is predicted to be particularly severe at midlatitudes and in the subtropics (Arnell 1999), with serious consequences for ecosystem and human health (Duda and El-Ashry 2000).

Water shortages often lead to conflicts between upstream users and downstream demands. When bodies of water cross jurisdictional boundaries, these conflicts are especially difficult to resolve and can present major challenges for resource management. For example, water in many regions of Africa is already scarce, and over 85% of the continent's water resources cross national borders, creating the potential for conflicts and a need for more integrated management (Ashton 2002). The Colorado River in the United States has the most complete water allocation of any river in the world (Christensen et al. 2004). However, climate change is predicted to lead to more droughts in the Colorado River basin, leading to more demand for water than the River will be able to supply (Christensen et al. 2004).

While the arid U.S. southwest has been coping with drought and interstate water allocation issues for over 80 years, these challenges are relatively nascent in the U.S. southeast (Ruhl 2005). Seasonal precipitation in the summer and winter has declined by nearly 10% in the eastern part of the region since the early 1900s, and the overall percentage of the U.S. southeast in drought has increased in recent decades (USGCRP 2009). Reduced precipitation in the southeast region, coupled with increases in societal demand for water, has resulted in water shortages that are leading to impacts on the economy and the ecosystems (USGCRP 2009).

**Figure 1:**
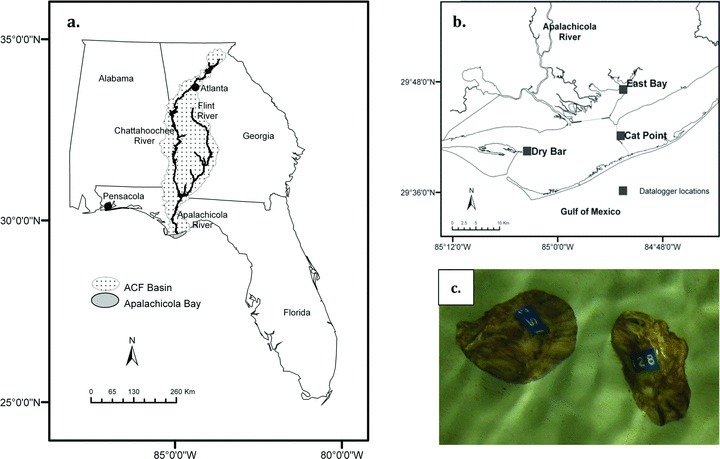
(a) Map of Apalachicola–Chattahoochee–Flint watershed. (b) Close-up of Apalachicola Bay, indicating locations of the sites East Bay, Cat Point, and Dry Bar. (c) Oysters (*Crassostrea virginica*) in the laboratory tank experiment.

The Apalachicola–Chattahoochee–Flint (ACF) River Basin, at 50,000 km^2^, is one of the larger contiguous watersheds in the United States, flowing through Georgia, Alabama, and Florida (Fig. 1a). Extensive water allocation and damming of the ACF River Basin began in the 1950s, and freshwater extraction has increased over time with population growth (USACE 1998). The U.S. Army Corps of Engineers (USACE) oversees water releases from the five major dams in the Chattahoochee River. Farthest upstream is Buford Dam (Buford, GA), forming Lake Sidney Lanier, which is currently the primary source of drinking water for the metropolitan Atlanta area. The Woodruff Dam (Chattahoochee, FL), forming Lake Seminole, is the farthest downstream dam and controls releases into the free-flowing Apalachicola River at the tristate border (FL, GA, and AL).

Approximately 7 million people reside in the ACF watershed, over 5 million of whom live in the metropolitan Atlanta, GA area (Ruhl 2005). From 2000 to 2006, metropolitan Atlanta exhibited the fastest growth rate of any U.S. city, according to the U.S. Census Bureau (2007). In 15 years (1982–1997), the population of the metropolitan Atlanta area increased by 60%, while simultaneously increasing its urbanized land by a disproportionate 80% (Fulton et al. 2001), making it one of the most “sprawling” cities in the U.S. (Burchfield et al. 2004). Such rapid changes in human population size and land use create challenges for maintaining adequate water quality and quantity (Karr 1991).

Approximately 2 billion liters of water per day are removed from the upper Chattahoochee River to supply Atlanta's drinking water, and additional removals occur in the lower Chattahoochee and Flint Rivers for agricultural irrigation (Ruhl 2005). As a result of increasing drought and water use in the ACF River Basin, interstate “water wars” have ensued, with Georgia fighting to withhold water in upstream reservoirs for municipal needs, and Alabama and Florida demanding water releases to fuel downstream agricultural and estuarine productivity (Ruhl 2005). These wars have resulted in court battles, extending through the level of U.S. District Court. However, after 20 years of litigation and failed attempts to create an inter-state compact, there is still no resolution (Ruhl 2005).

A severe regional drought occurred in the U.S. southeast in 2007, the second-driest year on record for Atlanta (CRS 2008). Reduced runoff into the ACF watershed led to the lowest levels ever recorded in Lake Lanier, with impacts on downstream reservoirs (CRS 2008). The USACE adopted the Interim Operating Plan (IOP) for ACF dam management in 2006, setting the minimum flow requirement for Woodruff Dam at 5000 cubic feet per second (cfs) to protect downstream threatened and endangered species in the Apalachicola River (CRS 2008). In November 2007, due to the severity and duration of the drought, the USACE began to manage the ACF dams under an Exceptional Drought Operations (EDO) amendment to the IOP, which decreased the minimum flow requirement to 4500 cfs at Woodruff Dam until June 2008 (CRS 2008).

There are serious concerns about the impacts of reduced freshwater input on the biologically rich and economically important habitats of the Apalachicola watershed (Fitzhugh and Richter 2004; Ruhl 2005). Many rare, endemic, threatened, and endangered plant and animal species reside within the Apalachicola River basin (Ziewitz 2005; Edmiston 2008). Downstream, the estuary is one of the more diverse and productive ecosystems in the world (Livingston 1991). The Apalachicola National Estuarine Research Reserve (ANERR) includes 1000 km^2^ of estuarine waters and provides critical foraging and nursery habitat for diverse fish and invertebrate assemblages that are commercially and recreationally harvested (Edmiston 2008). Salinity in Apalachicola Bay is highly dynamic and, depending on season and freshwater input, varies anywhere from almost entirely fresh (3 parts per thousand [ppt]) to entirely marine (33 ppt; Livingston et al. 2000). Under conditions of normal river flow, phytoplankton productivity is high in the estuary, supporting rapid growth rates of filter-feeding invertebrates, such as oysters (Livingston et al. 1997).

Estuarine oysters are ideal indicator organisms for studying the impacts of reduced freshwater input on ecosystem health due to their high abundance, sensitivity to changes in salinity, and economic and ecological importance. Apalachicola Bay produces 90% of the oysters (*Crassostrea virginica*) for human consumption in Florida and 10% of the oysters for the entire United States (USACE 1998). Over the past two decades, approximately 600–1100 oyster harvesters have worked at the Bay annually, resulting in average dockside oyster landings of 1.5–2 million pounds (∼680–900 metric tons) per year (Edmiston 2008). Commercial oyster harvesters rely on the river-dominated productivity of the estuarine environment, and resulting rapid growth rates and high reproductive output of Apalachicola oysters, for their income. Historically, the oyster industry has accounted for nearly half of the income of Franklin County (Whitfield and Beaumariage 1977). In addition to being economically important, oysters serve as valuable ecosystem engineers through modifying flow, filtering water, and enhancing diversity by providing three-dimensional habitat for hundreds of species (Wells 1961; Lenihan 1999; Coen and Grizzle 2007).

In recent decades, oyster populations have declined on the Gulf of Mexico and Atlantic Coasts of the United States, primarily due to a combination of overharvesting, disease, poor recruitment, and the loss of reef structural integrity (MacKenzie 1996; Seavey et al. 2011). Oyster population size, and therefore catch per unit effort, is negatively correlated with reduced freshwater input (Wilber 1992). Possible explanations for this pattern include sublethal stress effects of high salinity on growth and reproduction, as well as increased exposure to marine predators and diseases that would otherwise be kept in check by low salinity.

A major threat to oysters is “Dermo” disease (*Perkinsus marinus*), a protozoan parasite that is found from the Gulf of Mexico to Maine and thrives in high-salinity conditions. Transmission occurs when oysters ingest infective stages of parasites that are released into the water column from heavily infected oysters that are dying or decaying (Ragone Calvo et al. 2003). Infections can lead to reproductive suppression (Barber 1996) and/or mortality (La Peyre et al. 2003) in oyster hosts. Dermo is more virulent and lethal when salinity and water temperature are high in the summer and fall (Chu and Volety 1997) than in winter months, when cold water inhibits parasite activity (Chu and LaPeyre 1993). Most infections occur during an oyster's first year of life and become lethal in their second or third year (Ford and Tripp 1996). Therefore, larger, older oysters are predicted to be more susceptible to Dermo-related mortality than smaller oysters (Powell et al. 1997).

The objectives of this study were to:

- Investigate the effects of local (i.e., rainfall) and regional (i.e., upstream dam releases) freshwater input on estuarine salinity.
- Survey the health and condition of oyster populations during a severe and prolonged drought event.
- Test the effects of salinity and seasonality on oyster disease using controlled laboratory experiments.
- Provide information on salinity thresholds to inform water resource, estuarine, and fisheries management.

## Materials and Methods

### Environmental monitoring

Streamflow during the drought of 2007–2008 was compared to historical streamflow (from 1930 to 2008) using the U.S. Geological Survey (USGS) Streamflow Duration Hydrograph Builder (http://waterwatch.usgs.gov/index.php) for USGS station 02358000 (30°42′03″ N, 84°51′33″ W). This station is 1 km below Woodruff Dam, FL, at the confluence of the Chattahoochee and Flint Rivers at the GA–AL–FL tristate border, which is 173 km above the mouth of the Apalachicola River. This site serves as a good proxy for assessing the Apalachicola River's influence on coastal systems, as flow rates are highly correlated with downstream streamflow (Morey et al. 2009).

Temperature and salinity were recorded continuously (every 15 min) for two years (January 2007–December 2008) using YSI-6600 EDS sondes (YSI Inc., Yellow Springs, OH) maintained by the ANERR at three sites in Apalachicola Bay, FL (Fig. 1b):

- East Bay (29°47′8.88′ N, 84°52′30.72′ W), a site in the northeast of the Bay that typically exhibits lower salinity and is highly influenced by freshwater input;
- Cat Point (29°42′7.56″ N, 84°52′48.72″ W), a major oyster bar in the eastern Bay;
- Dry Bar (29°40′28.92″ N, 85°03′29.88″ W), a major oyster bar in the western Bay.

Rainfall was recorded continuously using a Tipping Bucket Rain Gauge (Model TE525, Campbell Scientific, Inc., Logan, UT) at a weather station maintained by ANERR in East Bay (29°47′27.24″ N, 84°53′0.24″ W). Discharge data from Woodruff Dam (30°42′29″ N, 84°51′47″ W) were made available by the USACE at: http://water.sam.usace.army.mil/acfframe.htm.

### Oyster disease surveys

Monthly surveys of oyster condition and disease were performed at Cat Point and Dry Bar, two of the major oyster reefs in Apalachicola Bay, from November 2007 to December 2008. Oysters (Fig. 1c) were collected at ∼1.8 m depth using hand tongs, which are iron rakes (∼1 m in width) attached to 4-m long wooden handles that are manually controlled to scissor and rake the bottom. At each of the two sites, 28–30 oysters of various sizes were collected, with the exception of the November 2007 sampling date, when only 18 oysters from Dry Bar and 20 oysters from Cat Point were collected. Oysters were cleaned on the boat, and all fouling organisms (primarily attached juvenile and adult oysters, barnacles, and mussels) were removed. The oysters were then shipped live to the Virginia Institute of Marine Science (VIMS) in Gloucester Point, VA, for condition and disease analyses as described in the “Condition and disease analyses” section.

### Oyster tank experiments

To evaluate the seasonal effects of salinity and temperature on disease in oysters, laboratory tank experiments were conducted twice: once in the winter and once in the summer. Oysters were collected using hand tongs at Dry Bar (February 7, 2008, for winter experiment, June 4, 2008, for summer experiment) and were cleaned and separated as described above. They were then transported on ice to the Florida State University Coastal & Marine Laboratory (FSUCML) in St. Teresa, FL for acclimation prior to experiment initiation.

Oysters were acclimated in a temperature-, light-controlled environmental chamber at FSUCML. Eighty oysters, 40 large (length >70 mm; range 70.1–108.1 mm) and 40 small (length <70 mm; range 36.3–70.0 mm) were placed into each of four acclimation tanks. For both the winter and summer experiments, oysters were acclimated for one week at ambient salinity (24 ppt in winter experiment, 30 ppt in summer experiment) and temperature (15°C in winter experiment, 25°C in summer experiment). These ambient conditions were based on the average temperature and salinity values in Apalachicola Bay as recorded by ANERR data loggers during the three to four weeks prior to acclimation for each experiment (winter and summer). During acclimation, oysters were fed 2 mL (∼9.2 billion cells of *Isochrysis galbana* 1800 culture (ISO QT; Reed Mariculture Inc., Campbell, CA) daily with a disposable Pastuer pipette.

Salinities for acclimation and experimental treatments were established by mixing fresh water (nonchlorinated well water) with sea water to achieve the desired treatment level (measured using a YSI-85 handheld instrument; YSI Inc., Yellow Springs, OH). Prior to addition to the tanks, water was filtered through 50, 10, and 1 μm mesh filter bags to remove particulate matter. Once tanks were filled, water in each tank was aerated using bubblers and filtered continuously with an aquarium pump connected with tubing to a PVC pipe (10.2 cm diameter) filled with crushed oyster shell and filter material. Filters were turned off for 4 h each day during feeding.

The following protocol was identical for both the winter and summer experiments. Salinity was manipulated as described above to achieve the following four treatments: 9 ppt, 17 ppt, 25 ppt, and 33 ppt (*n* = 4 tanks per treatment; 16 tanks total), spanning an evenly distributed, realistic range of estuarine salinity values. Temperature was held constant (at 15°C in winter experiment, 25°C in summer experiment) throughout the duration of each experiment, and lights were set on a daily 12 h:12 h light:dark cycle (light from 7:00 AM–7:00 PM).

On the first day of each experiment (February 14, 2008, for winter; June 11, 2008, for summer), oysters were removed from acclimation tanks, individually labeled with numerical tags, measured to the nearest 0.1 mm, and weighed to the nearest 0.1 g. They were then randomly assigned to one of the 16 tanks (*n* = 4 per salinity treatment), such that there were 10 large (>70 mm length) and 10 small (<70 mm length) oysters per tank.

Each tank was individually and continuously aerated and filtered as described above throughout the experiments. Each tank was provided with 0.5 mL (∼2.3 billion cells) of *I. galbana* 1800 culture daily, and filters were turned off for 4 h during feeding. Partial (50%) water changes were performed weekly, and water quality parameters (nitrite, nitrate, ammonium) were quantified several times throughout the experiment to ensure that the frequency of water changes was sufficient for maintaining clean water. Salinity was checked daily to confirm that tank salinity matched the desired treatment values; no tank deviated from its treatment value by more than 2 ppt throughout both experimental runs, and most remained within 1 ppt. Oyster mortality was quantified daily, and dead oysters were documented and removed.

At the end of five weeks (March 18, 2008, for winter; July 16, 2008, for summer), the experiments were terminated. From the surviving oysters in each tank, five large and five small individuals were randomly selected and shipped live to VIMS for condition and disease analyses.

### Condition and disease analyses

Condition index was assessed as an indicator of sublethal stress. Oysters with low condition indices have a lower ratio of tissue to shell, which can indicate low food availability, high disease load, and/or reduced reproductive potential. Oysters were dissected, tissues and shells were separated and weighed, and a wet-weight condition index was developed as follows:


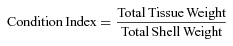


Dermo infection (presence/absence) and infection intensities were determined for each individual using Ray's Fluid Thioglycollate Medium (RFTM; Ray 1954) and microscopy (Olympus BX51; Olympus America Inc., Center Valley, PA). Weighted prevalence was then assigned to categories based on the number of parasite cells in the preparations of oyster host tissue, using a modified version of the semiquantitative scale of Ray (1954) as described in Table 1.

**Table 1:** Semi-quantitative scale for categorizing weighted prevalence of Dermo infection in oysters, modified from Ray (1954)

Weighted prevalence	Infection intensity	Description
0	No infection	No cells per preparation
0.5	Rare	One to two cells per preparation
1	Very light	3–10 cells per preparation
	Light	11–100 cells per preparation
2	Light-moderate	Some areas entirely free of parasites, and other areas show localized concentrations of ∼25–50 cells. In some cases, parasites are scattered uniformly throughout the preparation such that 2–3 are seen in each field at 100× magnification
3	Moderate	Parasites so numerous in the tissues that some can be found in every field at 100× magnification, although the masses of parasite cells are fairly localized
4	Moderate-heavy	Parasites present in large numbers. Less than one-half of the tissue appears blue macroscopically
5	Heavy	Parasites occur in enormous numbers. Major part of the tissue appears green–blue to blue–black in color macroscopically
	Very heavy	Parasites occur in enormous numbers. Entire tissue appears blue–black macroscopically

### Statistical analyses

All analyses were performed using JMP 7.0 statistical software (SAS Institute Inc., Cary, NC). Salinity in Apalachicola Bay (calculated as average daily salinity from East Bay, Cat Point, and Dry Bar), local rainfall (average daily rainfall for East Bay), and upstream dam discharge (average daily discharge from Woodruff Dam) were averaged for each month from January 2007 to December 2008. Linear regressions were performed on these monthly averages to analyze the relationships between Woodruff Dam discharge and downstream salinity for each site in Apalachicola Bay. In addition, regressions were conducted to determine the relationships between local rainfall and salinity at each Apalachicola Bay site.

For the oyster disease surveys, linear regressions were performed using monthly average data to investigate relationships between: temperature and condition index, salinity and condition index, temperature and weighted prevalence, salinity and weighted prevalence, temperature and salinity, and weighted prevalence and condition index for oysters at each of the two sites (Cat Point and Dry Bar).

For the laboratory experiment, three-way ANOVAs were conducted with season, salinity, oyster size, and all possible interactions as explanatory variables for the following separate response variables: mortality, condition index, and weighted prevalence. Tukey–Kramer honestly significant difference (HSD) post-hoc tests were performed to determine significant differences between pairwise comparisons. Data were transformed as follows to improve normality: mortality data were arcsin square root transformed, and condition index data and weighted prevalence data were natural log transformed. Regression analyses were performed using transformed averages from each tank to determine the linear relationships between (1) weighted prevalence and condition index, (2) condition index and mortality, and (3) weighted prevalence and mortality.

## Results

### Environmental monitoring

**Figure 2:**
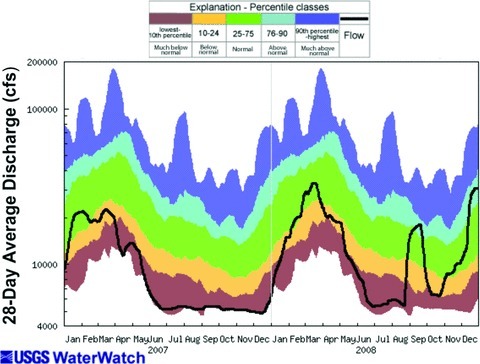
The 2007–2008 drought (black line, depicting flow) within the context of historical 28-day average discharge (from 1930 to 2008) developed using the USGS Streamflow Duration Hydrograph Builder (http://waterwatch.usgs.gov/index.php) for USGS station 02358000, 1 km below Woodruff Dam.

Comparisons of streamflow data from 2007 to 2008 to historical streamflow over an 88-year period indicated the severity of the drought event (Fig. 2). During the majority of 2007 and for several months of 2008, streamflow fell far below normal, with prolonged periods in the 10th percentile, and reaching near-minimum or minimum flow rates on multiple occasions (Fig. 2).

**Figure 3:**
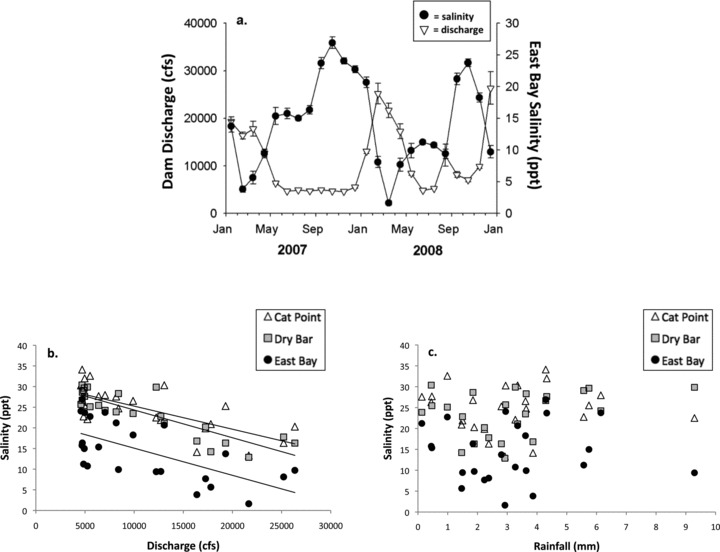
(a) Monthly average salinity from East Bay in Apalachicola Bay and monthly average dam discharge at Woodruff Dam. Y-error bars indicate standard error. (b) Scatterplot of monthly average salinity from East Bay, Dry Bar, and Cat Point in Apalachicola Bay versus monthly average dam discharge at Woodruff Dam. (c) Scatterplot of monthly average salinity from East Bay, Dry Bar, and Cat Point in Apalachicola Bay versus monthly average local rainfall in Apalachicola.

There was a strong relationship between Woodruff Dam discharge and Apalachicola Bay salinity at each of the three sites (Fig. 3a and b), which can be described using the following equations:


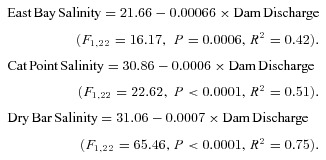


In contrast, there was no relationship between local rainfall and Apalachicola Bay salinity at any of the three sites (Fig. 3c): East Bay (*F*_1,22_ = 0.001, *P* = 0.97, *R*^2^ = 0.00), Cat Point (*F*_1,22_ = 0.008, *P* = 0.93, *R*^2^ = 0.0004), or Dry Bar (*F*_1,22_ = 2.21, *P* = 0.15, *R*^2^ = 0.09).

### Oyster disease surveys

**Figure 4:**
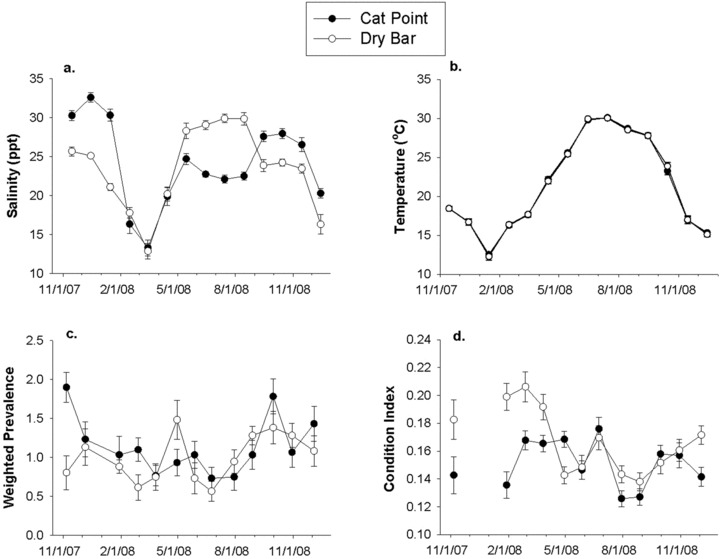
Oyster survey data from Cat Point and Dry Bar. Monthly average salinity (a), monthly average temperature (b), Dermo weighted prevalence (c), and condition index (d) from November 2007 to December 2008. Y-error bars indicate standard error.

Average salinity between November 2007 and December 2008 ranged from 13.3 + 1.0 ppt to 32.6 + 0.6 ppt at Cat Point and from 12.9 + 1.0 ppt to 29.9 + 0.5 ppt at Dry Bar (Fig. 4a). Average temperature was very similar in timing and magnitude at both sites, ranging from 12.5 + 0.4°C to 30.1 + 0.2°C at Cat Point and from 12.3 + 0.5°C to 30.0 + 0.1°C at Dry Bar (Fig. 4b). The percentage of oysters infected with Dermo was high during each sampling period, ranging from 53.3% to 95.0% at Cat Point and from 53.3% to 93.3% at Dry Bar.

Cat Point weighted prevalence ranged from 0.73 + 0.14 to 1.90 + 0.19, and Dry Bar weighted prevalence ranged from 0.57 + 0.13 to 1.48 + 0.25 (Fig. 4c). There was no relationship between temperature and weighted prevalence at either Dry Bar (F_1,11_ = 0.30, *P* = 0.60, *R*^2^ = 0.03) or Cat Point (*F*_1,11_ = 0.60, *P* = 0.45, *R*^2^ = 0.05). There was a slight (but statistically insignificant) positive relationship between salinity and weighted prevalence at Cat Point (*F*_1,11_ = 3.42, *P* = 0.09, *R*^2^ = 0.24); there was no relationship at Dry Bar (*F*_1,11_ = 0.02, *P* = 0.89, *R*^2^ = 0.002).

Condition index ranged from 0.13 + 0.00 to 0.18 + 0.01 at Cat Point and from 0.14 + 0.01 to 0.21 + 0.01 at Dry Bar (Fig. 4d). In Dry Bar oysters, there were negative relationships between temperature and condition index (*F*_1,10_ = 15.58, *P* = 0.003, *R*^2^ = 0.61), as well as between salinity and condition index (*F*_1,10_ = 6.16, *P* = 0.03, *R*^2^ = 0.38). A positive relationship was found between temperature and salinity at Dry Bar (*F*_1,11_ = 12.29, *P* = 0.005, *R*^2^ = 0.53), and a period of both high salinity and high temperature from May through September 2008 coincided with low condition index (Fig. 4a, b, and d), suggesting potential synergistic effects of salinity and temperature. No relationships were found with either temperature (*F*_1,10_ = 0.02, *P* = 0.88, *R*^2^ = 0.002) or salinity (*F*_1,10_ = 1.67, *P* = 0.23, *R*^2^ = 0.14) and condition index for Cat Point oysters. In contrast to Dry Bar, there was no relationship between temperature and salinity at Cat Point (*F*_1,11_ = 0.03, *P* = 0.87, *R*^2^ = 0.002), and there were no periods when both high salinity and high temperature coincided (Fig. 4a and b). In general, there was less variability in condition index throughout the sampling period at Cat Point than at Dry Bar (Fig. 4d).

There was a negative relationship between weighted prevalence and condition index for oysters at Dry Bar (*F*_1,10_ = 6.37, *P* = 0.03, *R*^2^ = 0.39); there was no relationship at Cat Point (*F*_1,10_ = 0.22, *P* = 0.65, *R*^2^ = 0.02).

### Oyster tank experiments

**Figure 5:**
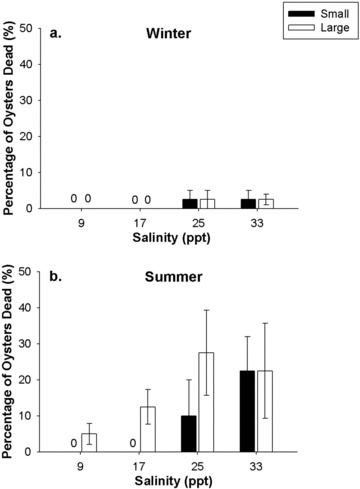
Oyster mortality in the winter (a) and summer (b) tank experiments. Y-error bars indicate standard error.

Oyster mortality was higher overall in the summer experiment than in the winter experiment (*F*_1,48_ = 21.73, *P* < 0.0001, Table 2, Fig. 5), demonstrating a strong seasonal effect. In addition, mortality was higher in general for large oysters than small oysters (*F*_1,48_ = 4.30, *P* = 0.04), indicating size-specific responses, and was highest overall for large oysters in the summer (*F*_1,48_ = 4.30, *P* = 0.04). Mortality was highest at 33 ppt, intermediate at 25 ppt, and lowest at 17 and 9 ppt (*F*_3,48_ = 5.23, *P* = 0.003), indicating that oyster mortality increases with high salinity.

**Table 2:** Results of three-way ANOVAs testing effects of season, salinity, oyster size, and all possible interactions on arcsin-square-root transformed mortality, ln-transformed condition index, and ln-transformed weighted prevalence. Bolded numbers indicate statistical significance (*P* < 0.05)

	Mortality				Condition index				Weighted prevalence			
			
Parameter	df	SS	*F*	*P*	df	SS	*F*	*P*	df	SS	*F*	*P*
Season	1	0.76	21.73	**<0.0001**	1	3.04	153.33	**<0.0001**	1	15.79	73.42	**<0.0001**
Salinity	3	0.55	5.23	**0.003**	3	0.10	1.71	0.18	3	1.26	1.96	0.13
Oyster size	1	0.15	4.30	**0.04**	1	0.32	16.03	**0.0002**	1	0.93	4.31	**0.04**
Salinity × Season	3	0.19	1.80	0.16	3	0.20	3.34	**0.03**	3	0.35	0.53	0.66
Salinity × Oyster size	3	0.10	0.99	0.41	3	0.05	0.85	0.47	3	0.15	0.24	0.87
Season × Oyster size	1	0.15	4.30	**0.04**	1	0.004	0.18	0.67	1	1.43	6.65	**0.01**
Season × Salinity × Oyster size	3	0.10	0.99	0.41	3	0.07	1.15	0.34	3	0.02	0.03	0.99
Error	48	2.00			48	0.95			48	10.32		
Total	63	3.67		**0.0002**	63	4.72		**<0.0001**	63	30.25		**<0.0001**

**Figure 6:**
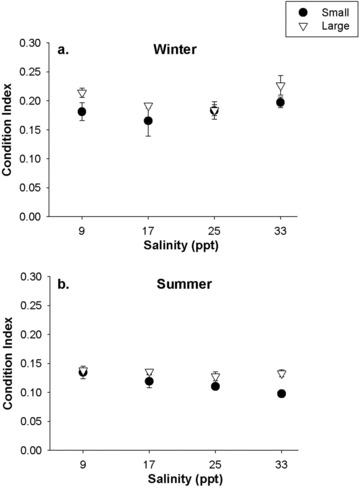
Oyster condition index (tissue weight/shell weight) in the winter (a) and summer (b) tank experiments. Y-error bars indicate standard error.

Condition index of oysters was higher in the winter than in the summer (*F*_1,48_ = 153.33, *P* < 0.0001, Table 2, Fig. 6). Large oysters had higher condition indices than did small oysters (*F*_1,48_ = 16.03, *P* = 0.0002). There was no effect of salinity on condition index (*F*_3,48_ = 1.71, *P* = 0.18).

**Figure 7:**
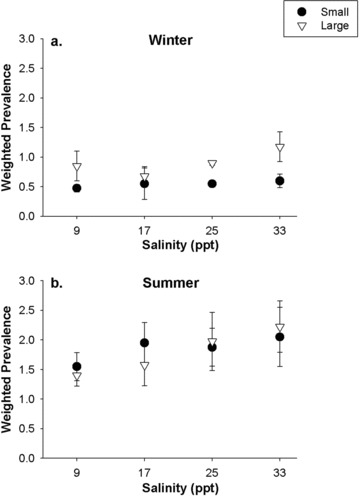
Oyster weighted prevalence in the winter (a) and summer (b) tank experiments. Y-error bars indicate standard error.

Weighted prevalence of Dermo disease in oysters was higher in the summer than in the winter (*F*_1,48_ = 73.42, *P* < 0.0001, Table 2, Fig. 7), indicating that severity of disease was higher in the warmer months. Large oysters had higher weighted disease prevalence than did small oysters (*F*_1,48_ = 4.31, *P* = 0.04), which may have contributed to size-specific mortality. Oysters of both size classes in the summer had higher weighted prevalence than did large oysters in the winter, which had higher weighted prevalence than small oysters in the winter (*F*_1,48_ = 6.65, *P* = 0.01). There was no effect of salinity on weighted prevalence (*F*_3,48_ = 1.96, *P* = 0.13).

There were negative relationships between weighted prevalence and condition index (*F*_1,62_ = 28.43, *P* < 0.0001, *R*^2^ = 0.31), as well as between condition index and mortality (*F*_1,62_ = 8.92, *P* = 0.004, *R*^2^ = 0.13). In addition, there was a positive relationship between weighted prevalence and mortality (*F*_1,62_ = 12.69, *P* = 0.0007, *R*^2^ = 0.17).

## Discussion

The synergistic effects of temperature and salinity are among the more dominant factors affecting the biology of estuarine organisms (Shumway 1996). Results of this study showed that oyster mortality was strongly linked to both salinity and seasonal temperature. In the winter, when Dermo (*P. marinus*) typically remains dormant due to cold water temperatures, mortality in the experiment was low at all salinities. The synergistic effects of low temperature and low salinity have been shown to reduce Dermo cell viability (La Peyre et al. 2010). In the summer experiment, in temperature conditions favoring parasite proliferation, mortality was highest at high salinity (33 ppt), intermediate at 25 ppt, and the lowest in low salinity (17 and 9 ppt). These findings are consistent with previous studies (e.g., Chu et al. 1994; Chu and Volety 1997), which indicated that Dermo-related impacts are maximized when both temperature and salinity are elevated.

A negative relationship was documented between Dermo weighted prevalence and condition index in both the experiment and in the field survey (for Dry Bar oysters), which aligns with previous findings that Dermo infection can negatively affect oyster condition index (e.g., Dittman et al. 2001) and reproduction (e.g., Barber 1996). Reduced oyster condition has implications for the seafood industry, as economic value is often assigned to the weight of shucked tissue. In addition, low condition indices often correspond with reduced reproductive potential (i.e., less reproductive tissue and therefore fewer viable gametes), having potential consequences for future oyster population sizes.

Oysters in the experiment exhibited size-specific mortality, as large oysters died more frequently than small oysters, particularly in the summer. No mortality of small oysters occurred in either season at salinities below 25 ppt. In contrast, some level of mortality was exhibited for large oysters at all salinities in the summer experiment, with ∼20–30% mortality at 25 and 33 ppt. These mortality rates are likely lower than the mortality rates that occurred in Apalachicola Bay during the severe and prolonged drought event of 2007–2008, as the laboratory exposure to high-salinity conditions lasted only five weeks. Field observational data confirmed that the five-week laboratory exposures were conservative compared to high-salinity conditions on Apalachicola Bay oyster bars. When Woodruff Dam was set at minimum flow (5000 cfs) from June to December 2007 to retain water for upstream use, average monthly salinity at Cat Point and Dry Bar remained above 25 ppt for the entire 7-month duration. In addition, daily average salinity at Cat Point remained at or above 33 ppt for an entire seven-week period from September 18 to November 3, 2007, with the exception of two 1-day decreases (to 27 and 32 ppt) and one 4-day decrease (salinities ranging from 24 to 31 ppt). This evidence indicates that oysters in Apalachicola Bay experience chronic exposure to high-salinity water during drought, placing them at high risk of disease-related mortality. It is also likely that these high-salinity conditions led to reduced growth, given that salinity exceeded optimal ranges for oyster growth (estimated at 20–25 ppt for Cat Point and 17–26 ppt for Dry Bar; Wang et al. 2008).

Disease-related effects of high salinity on oyster growth and mortality can impact the success of oyster fisheries. The legal size limit for oyster harvest in Apalachicola Bay is 76 mm (Florida FWC Regulation CH 46–27), which is similar to the experiment's “large” size class (>70 mm) and a size that Apalachicola oysters typically reach within 18 months (Ingle and Dawson 1952). Oysters become susceptible to mortality from Dermo infection in only their second year of life (Ford and Tripp 1996). Therefore, reduced growth rates resulting from high-salinity conditions means that many oysters may not reach harvestable size before dying from Dermo disease. This causes challenges for fisheries management and fishery-dependent economies. Oyster declines also lead to the loss of valuable ecosystem services, such as water filtration, food for predators, and the provision of habitat for a diversity of reef-associated species.

In addition to impacts on oysters, other ecosystem-level consequences were documented in ANERR during the 2007 drought event. Declines were observed in the tape grass, *Vallisneria americana*, within the lower Apalachicola River and its distributaries (ANERR, unpubl. data), resulting in the loss of habitat for vegetation-dependent fish and invertebrates. In Apalachicola Bay, reductions occurred in species typically found in shallow, lower-salinity waters, including shads, pogies, Gulf and southern flounders, mullet, sheepshead, and redfish (ANERR, unpubl. data). In contrast, increases were found for species that use high-salinity, seagrass areas, such as pinfish, pigfish, jennies, lizardfish, silver perch, and snappers (ANERR, unpubl. data). Species typically found offshore began to appear in the Bay and over the seagrass beds behind the Bay's barrier islands (ANERR, unpubl. data). In general, shifts in composition occurred, with higher-salinity species moving into habitats that are typically dominated by lower-salinity species. Reduced nutrient input from the Apalachicola River during low-flow years is hypothesized to lead to declines in offshore productivity, with consequences for commercially important fisheries (Morey et al. 2009). These changes can influence trophic relationships, other species interactions, and ecosystem function, as well as the economies and societies that depend on healthy ecosystems for their livelihoods and well-being.

The results of this study have important implications for water resource management. Upstream releases of freshwater during prolonged periods of high temperature and high salinity could potentially be used to help ameliorate disease-related mortality in downstream oyster populations (La Peyre et al. 2009). The laboratory experiment revealed a possible “tipping point” between salinity values of 17 and 25 ppt, at which prolonged exposure increases mortality rates of large oysters and becomes lethal for small oysters. It is possible that summertime freshwater releases, if the magnitude and timing were carefully calculated, could substantially reduce stress for oysters and other estuarine organisms. This would require a more integrated watershed management approach that explicitly incorporates downstream ecosystem health into upstream policy and decision-making processes.

Climate variability, climate change, and human population growth exacerbate the existing challenges for sustainable water, coastal, and marine resource management (Vörösmarty et al. 2000; Christensen et al. 2004; Tompkins and Adger 2004). The prolonged drought event that occurred during this study was associated with a moderate La Niña. During La Niña events, U.S. southeastern states are typically warmer and drier (Philander 1989), creating conditions favoring Dermo proliferation (Soniat et al. 2009), and Apalachicola River flow is anomalously low (Morey et al. 2009). Increases in the frequency and severity of regional drought events are predicted for the U.S. southeast under climate change scenarios (USGCRP 2009). In addition, population growth in this region continues to be rapid. Between 2000 and 2010, the south accounted for 54.4% of the U.S. population growth, and the metropolitan Atlanta area grew by 24.0% (Mackun and Wilson 2011). These pressures will lead to additional constraints on resources that are already limited, particularly during drought.

The U.S. southeast region serves as a microcosm of global sustainability issues. Over 1 billion people currently live in areas that are likely to require management intervention because of water stress by the year 2050 (Palmer et al. 2008). To meet the demands of the coming decades, there is an urgent need for more coordinated and adaptive water resource management that integrates considerations of the valuable ecosystem services on which humans depend (Duda and El-Ashry 2000; Milly et al. 2008).
